# *Crocus sativus* L. Stigmas, Tepals, and Leaves Ameliorate Gentamicin-Induced Renal Toxicity: A Biochemical and Histopathological Study

**DOI:** 10.1155/2022/7127037

**Published:** 2022-10-01

**Authors:** Sabir Ouahhoud, Noureddine Bencheikh, Amine Khoulati, Salma Kadda, Samira Mamri, Anas Ziani, Sanae Baddaoui, Fatima-Ezzahra Eddabbeh, Soufiane Elassri, Iliass Lahmass, Redouane Benabbes, Mohamed Addi, Christophe Hano, Mohammed Choukri, Amal Bennani, Abdeslam Asehraou, Ennouamane Saalaoui

**Affiliations:** ^1^Laboratory of Bioresources, Biotechnology, Ethnopharmacology and Health, Faculty of Sciences, Université Mohamed Premier, Oujda 60000, Morocco; ^2^Laboratory of Improvement of Agricultural Production, Biotechnology, and Environment, Department of Biology, Faculty of Sciences, Université Mohamed Premier, Oujda 60000, Morocco; ^3^Central Laboratory Service - CHU, Mohammed VI, Faculty of Medicine and Pharmacy, University Mohamed Premier, Oujda 60000, Morocco; ^4^Laboratory of Biotechnology, Environment, Agri-food and Health, Faculty of Science Dhar Mahraz, Sidi Mohamed Ben Abdallah University, Fez 1796, Morocco; ^5^Laboratoire de Biologie des Ligneux et des Grandes Cultures, INRA USC1328, Orleans University, CEDEX 2, Orléans 45067, France

## Abstract

The most costly spice in the world, *Crocus sativus* L. (*C. sativus*), has been used for more than 3,000 years. It has various beneficial applications in a range of fields, including aromas, colorants, and medications, but its usefulness as a food flavoring and coloring ingredient is the highest. Large quantities of by-products from the processing of saffron are typically thrown as unwanted bio-residues. This study's goal was to assess and compare the nephroprotective effects of hydroethanolic extracts of *C. sativus* stigmas, tepals, and leaves on gentamicin (GM)-induced nephrotoxicity in rats. For that, we used a biochemical and histological investigation to propose new pharmaceutical valorizations. Based on the biochemical and histological analyses, it is concluded that all the studied parts of *C sativus* showed a renoprotective effect. Markedly, tepals revealed the most significant reduction of relative liver weight (*p* < 0.05), water intake (*p* < 0.05), plasma creatinine (*p* < 0.01), plasma urea (*p* < 0.01), plasma uric acid (*p* < 0.05), urinary protein (*p* < 0.01) and albumin (*p* < 0.001), and renal malondialdehyde (MDA) (*p* < 0.001). In addition, *C. sativus* tepals caused a significant increase in body weight (*p* < 0.05), urinary creatinine (*p* < 0.01), creatinine clearance (*p* < 0.05), and urinary urea (*p* < 0.05) compared with the gentamicin untreated (GM) group. This is confirmed by the histopathological study which shows that treatment with stigmas, tepals, and leaves preserved kidney morphology at the glomerular and tubular cell level. The studied extracts exhibit good recovery potential for nephrotoxicity induced by gentamicin. In order to create potent dietary supplements or phytomedicines, it would also be very interesting to confirm these actions through clinical research.

## 1. Introduction

Since more than 3000 years ago, saffron has been used as a spice. It is a part of the Iridaceae family and the *Crocus* genus, which has roughly 80 species. Saffron, the priciest spice in the world is mainly found in the Mediterranean and Southwest Asia [1]. Since ancient times, saffron has been used in many fields, including cosmetics, colorants, and medicines, although it is most commonly employed as a food flavoring and coloring ingredient [2]. Recent research have revealed the antioxidant [3], antitumor [4], antigenotoxic [5], antidiabetic [6], hepatoprotective [7, 8], and nephroprotective [9] properties of saffron stigma. Large volumes of by-products from the manufacture of saffron are typically thrown as unwanted bio-residues [10]. In order to get just 1 kg of the dry stigmas, approximately 63 kg of flowers, 1500 kg of leaves, and hundreds of defective or undersized bulbs are destroyed [11]. The profitability and sustainability of saffron production would significantly boost with the usage of these bioactive substances discovered in this organic waste [11]. A common antibiotic known as an aminoglycoside called gentamicin is used to treat severe infections brought on by aerobic bacteria [12]. One of the most significant side effects of aminoglycoside antibiotics, which can occur in 10–20% of patients, is renal impairment [13, 14]. Researchers have reported that aminoglycosides are antibiotics capable of causing nephrotoxicity by producing oxidative stress [15, 16]. This study's goal was to assess and compare the nephroprotective effects of extracts of *C. sativus* from the Moroccan Taliouine region on rat which the nephrotoxicity caused by gentamicin (GM). We performed a biochemical and histological investigation to offer novel valorizations, particularly in the pharmaceutical industry.

## 2. Materials and Methods

### 2.1. Chemicals

All of the chemicals utilized in this study were analytical-grade materials that were provided by Sigma Chemicals (MA, USA).

### 2.2. Plant Material

The *C. sativus* plant's stigmas, tepals, and leaves were gathered at a farm near Taliouine (30°31′54″ north, 7° 55′ 25″ west, Southern Morocco). Saffron was grown naturally in this area without the use of chemicals. The plant's various components were gathered between October and November of 2016. Botanical professor Fennane Mohammed of the Scientific Institute of Rabat, Morocco, determined the plant's botanical classification. Under the voucher code HUMPOM210, three species of the plant have been identified in the herbarium of the University Mohammed First in Oujda, Morocco.

### 2.3. Plant Material Preparation

Preparation of the plant material was done using the technique outlined by Ouahhoud et al. [8]. First, the stigmas and various *C. sativus* components were manually separated. The stigmas were indeed dried in an oven for four hours at 37°C. Tepals and leaves were dried in an oven for 24 hours each at 37°C. An electric mill was then used to grind the dry plant material.

### 2.4. Making Hydroethanolic Saffron Extracts

According to our early research, 80% ethanol was the solvent with the best biological characteristics [6, 8]. The Ouahhoud et al. [6] procedure is the one that is in use for this study. For 24 hours, stirring was done at ambient temperature and in the darkness, while the vegetable material was macerated in an 80/20 (v/v) ethanol/water mixture. The extraction ratio was 2 g of plant powder to 50 ml of solvent. The solvent was then filtered (0.45 m) after the first extraction, and the marc was collected for the subsequent extraction. Following three further iterations of this procedure, a rotary evaporator (at 40°C) was used to dry the entire extract phase.

### 2.5. Animals

Male Wistar rats those were nine-week-old and weighed 200–250 g were kept in the animal house of the Biology Department at the Faculty of Sciences in Oujda, Morocco, where they were subjected to a 12-hour light/dark cycle. Water and food were freely available to the rats [8].

### 2.6. Ethical Approval

The Faculty of Sciences, University Mohammed Premier, Oujda (01/21-LBBEH-04 and 15 March, 2021) approved the study, which was carried out in accordance with the US National Institutes of Health's recommendations. All of the animal experiments that were conducted during this investigation complied with the globally recognized manual for the handling and use of laboratory animals.

### 2.7. Plant Extracts Administration

The dry extracts (hydroethanolic) of the by-products of *C. sativus* are dissolved in distilled water. The administration of the stigma (STG), tepal (TPL), and leaf (LV) extract is performed orally using an intragastric tube for rats. The concentrations that would be employed were selected based on our preliminary research carried out in our laboratory [6, 8].

### 2.8. Animal Distribution and Experimental Protocol

Thirty male rats (nine-week-old, weight 200–250 g) were randomly divided into 5 groups of 5 rats which are as follows: N Group: normal control rats received daily, by intraperitoneal injection, physiological water and 1 hour later, by gavage, distilled water for 14 days of the study; GM group: intoxicated control rats receiving daily, by intraperitoneal injection, 80 mg/kg of gentamicin and 1 hour later, by gavage, distilled water for 14 days; STG group: intoxicated control rats receiving daily, intraperitoneally 80 mg/kg of gentamicin, and 1 hour later, orally, 50 mg/kg of the hydroethanolic extract of the stigmas for 14 days; TPL group: intoxicated control rats receiving daily, intraperitoneally 80 mg/kg of gentamicin, and 1 hour later, orally, 250 mg/kg of the hydroethanolic extract of tepals for 14 days; and LV group: intoxicated control rats receiving daily, intraperitoneally 80 mg/kg of gentamicin, and 1 hour later, orally, 250 mg/kg of the hydroethanolic extract of the leaves for 14 days. Urine was collected using metabolic cages for a full 24 hours after the treatment was finished. Both before and after the trial, the animals are weighed. The rats underwent pentobarbital anesthesia after receiving the therapy for 14 days. Blood is drawn from the abdominal aorta into heparin-filled tubes. The samples were then centrifuged for 10 min at 3000 rpm. The plasma was recovered and stored at −20°C for biochemical tests. The right kidney was then removed, rinsed with physiological water (NaCl 0.9%), and weighed. Finally, it was stored at −20°C for malondialdehyde (MDA) determination. The left kidney is rinsed with physiological water (0.9% NaCl), weighed, and stored in 10% formalin (fixation) for histological study.

### 2.9. Relative Kidney Weight

The following formula was used to determine the relative kidney weight:

(1). $$\begin{array}{c} \text{Relative weight of the kidneys}\left(\%\right)=\frac{\text{Absolute kidney weight}\left(g\right)X100}{\text{Body weight of the rat}\left(g\right)}. \end{array}$$

### 2.10. Histological Analysis

The purpose of this histological analysis (stained with hematoxylin and eosin) is to explore the structure of the renal tissue, the constitutive and functional relationships between their functional elements, and the turnover of the tissue. It is performed in several steps according to the protocol described by Hewitson and Darby [17].

### 2.11. Biochemical Tests Performed

In this study, the authors determined creatinine, urea, uric acid, and electrolyte (Na+, K+, and Cl−) plasma levels. Also, the urinary levels of creatinine, total protein, albumin, and uric acid were measured, and the creatinine clearance was calculated. Biochemical analysis was realized on a COBAS INTEGRA autoanalyzer (Roche Diagnostics) at the Mohammed VI Hospital in Oujda.

### 2.12. Creatinine Clearance

Based on plasma and urine creatinine concentrations, the following formula was used to determine creatinine clearance in order to evaluate the glomerular filtration rate:

(2) $$\begin{array}{c} \text{Creatinine clearance}\left(\frac{ml}{\min Xkg}\right)=\frac{\text{Urine creatinine}\left(mg/ml\right)X\text{ Urine flow rate}\left(ml/\min\right)X1000}{\text{Plasma creatinine}\left(mg/ml\right)X\text{ rat weight}\left(kg\right)}. \end{array}$$

The urine flow rate was calculated using the following formula:

(3) $$\begin{array}{c} \text{Urine flow rate}\left(\frac{ml}{\min}\right)=\frac{\text{urine volume value}\left(24h\right)}{1440\left(60\min X24h=1440\right)}. \end{array}$$

### 2.13. Malondialdehyde (MDA) Determination

A method developed by Ouahhoud et al. was used to measure lipid peroxidation [8]. For this, 0.5 g of the kidney was homogenized in a Potter tube with 5 ml of PBS (pH 7.4) at 0°C (ice). After that, the homogenate was centrifuged for 15 minutes at 14500 rpm. 2 ml of reagent (consisting of 0.375% TBA (thiobarbituric acid) and 15% TCA (trichloroacetic acid) that were dissolved in 0.25 N hydrochloric acid) were added with one milliliter of supernatant. The mixture was centrifuged at 4750 rpm for 5 minutes after being boiled in water for 30 minutes. The supernatant's absorbance was calculated at 535 nm. The amount of MDA is determined using the molar extinction coefficient of MDA (*ε*=1.56 × 10^5^M^−1^cm^−1^). Values were expressed as nmol of MDA per mg of the tissue.

### 2.14. The Qualitative Analysis of *C. sativus* Extract

The hydroethanolic extracts from *C sativus* was analyzed by high-performance liquid chromatography (Waters AllianceTM e2695 XC HPLC system outfitted with a 2998 Photodiode Array Detector. Samples (10 mg/ml) were injected onto a reversed-phase C18 column (5 *μ*m, 250 mm × 4.6 mm) at a flow rate of 1 mL/min. The following gradient of binary solvents (2% of acetic acid in water (solvent A) and methanol (solvent B)) was used for elution: initial 80% A and 20% B; 20 min 100% B; 25 min 100% B; 30 min 50% A and 50% B; 35 min 80% A and 20% B. The UV detection was carried out in the 220–575 nm range with an injection volume of 20 L. By comparing the retention period and maximum wavelength with reference materials and standards, the active compounds were found.

### 2.15. Statistical Analysis

With *n* = 5 for each group, the data were presented as mean SEM. The results of the data's normality test showed that all of the data had a normal distribution. One-way ANOVA was used to statistically analyze using the GraphPad Prism 5.0 program. With significant levels of *p* < 0.05, *p* < 0.01 and *p* < 0.001, Tukey's multiple comparison posttest was used to examine differences between the treatment groups.

## 3. Results

### 3.1. The Effect of Gentamicin and Extracts of Different Parts of *C. sativus* on Renal Histology

**Figure 1:**
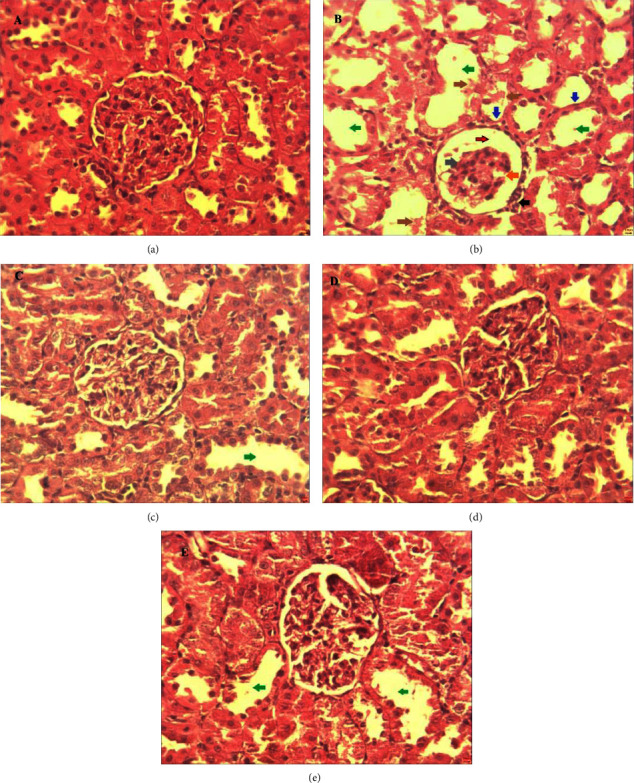
Effect of extracts from stigmas (50 mg/kg), tepals (250 mg/kg), and leaves (250 mg/kg) of *C. sativus* on histological changes of the kidney ((a), (b), (c), (d), and (e)) in gentamicin-intoxicated rats. Histological sections of the kidney tissue (stained with hematoxylin and eosin) were taken from the normal group (a), the gentamicin + distilled water group (b), the gentamicin + STG group (c), the gentamicin + TPL group (d), the and gentamicin + LV group (e). Glomerular degeneration (orange arrow), necrosis at Bowmen's capsule (gray arrow), distinction of Bowman's space (red arrow), mononuclear cell infiltration (black arrow), renal tubule desquamation (brown arrow), renal tubule atrophy (blue arrow), and renal tubule dilatation (green arrow).

Figure 1 shows the effect of extracts from different parts of *C. sativus* on the histological changes of the kidney (A, B, C, D, and E) in gentamicin-intoxicated rats.

### 3.2. The Effect of Gentamicin and *C. sativus* Extracts on Body Weight and Relative Kidney Weight

**Figure 2:**
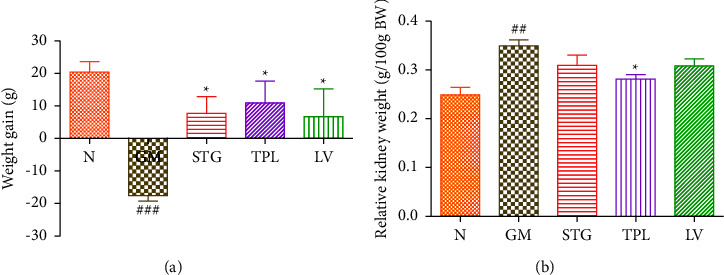
Effects of gentamicin and *C. sativus* extracts of stigmas (50 mg/kg), tepals (250 mg/kg), and leaves (250 mg/kg) on weight gain (a) and relative kidney weight (b) in rats. Values were expressed as mean ± SEM. (*n* = 5); ^###^*p* < 0.001 and ^##^*p* < 0.01 in comparison with the N group;  ^*∗*^*p* < 0.05 in comparison with the GM group.

The effects of hydroethanolic extracts of the stigmas, tepals, and leaves of *C. sativus* on gaining weight and relative kidney weight in rats with gentamicin nephrotoxicity are shown in Figures 2(a) and 2(b). In comparison to normal rats, the daily injection of gentamicin (80 mg/kg) caused a considerable drop in body weight (*p* < 0.001). However, daily administration of stigma, tepal, and leaf extracts significantly (*p* < 0.05) protected gentamicin-intoxicated rats against weight loss compared to untreated rats (GM group). Rats given GM showed a significant increase in relative kidney weight (*p* < 0.01) as compared to control rats. Rats given tepal extract treatment had a significant lower in relative kidney weight (*p* < 0.05) than the GM group. However, there was no discernible decrease in relative kidney weight between the GM group, stigma, and leaf extracts.

### 3.3. The Effect of Gentamicin and *C. sativus* Extracts on Water Intake and Urine Volume

**Figure 3:**
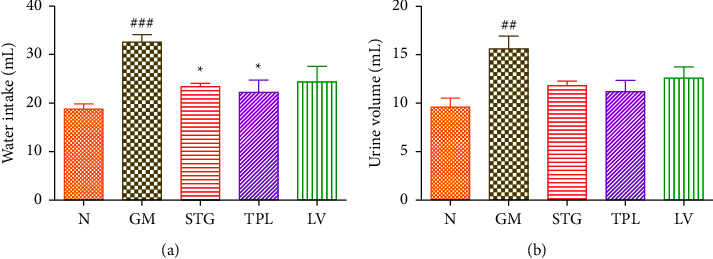
Effect of extracts from stigmas (50 mg/kg), tepals (250 mg/kg), and leaves (250 mg/kg) of *C. sativus* on water intake (a) and urine volume (b) in gentamicin-intoxicated rats. Values were expressed as mean ± SEM. (*n* = 5); ^###^*p* < 0.001 and ^##^*p* < 0.01 in comparison with the N group;  ^*∗*^*p* < 0.05 in comparison with the GM group.

Figure 3 shows the effect of hydroethanolic extracts of some *C. sativus*by-products on water intake and urine volume in gentamicin-intoxicated rats. The gentamicin-treated group showed a significant increase in water intake (*p* < 0.001) and urine volume (*p* < 0.01) compared with the normal group. Administration of the stigma and tepal extracts significantly (*p* < 0.05) decreased water intake compared to gentamicin-intoxicated rats (GM group). However, compared to the GM group, administration of the leaf extract had no discernible impact on the group's water consumption. In comparison to the GM group, the administration of the stigma, tepal, and leaf extracts had no significant influence on the urine volume.

### 3.4. The Effect of Gentamicin and *C. sativus* on Plasma Creatinine, Urinary Creatinine, and Clearance

**Figure 4:**
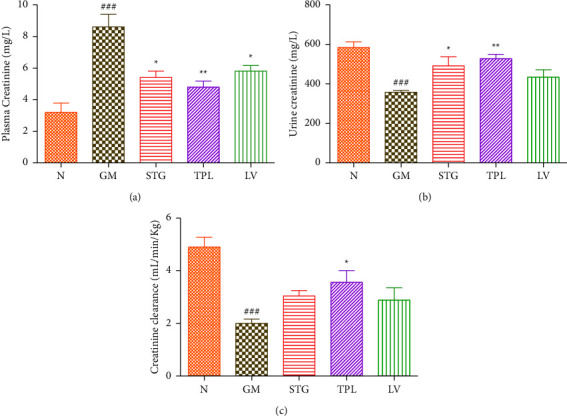
The effect of gentamicin and extracts of *C. sativus* stigmas (50 mg/kg), tepals (250 mg/kg), and leaves (250 mg/kg) on plasma creatinine (a), urine creatinine (b), and creatinine clearance (c) in rats. Values were expressed as mean ± SEM. (*n* = 5); ^###^*p* < 0.001 in comparison with the N group;  ^*∗*^*p* < 0.05 and  ^*∗∗*^*p* < 0.01 in comparison with the GM group.

Figures 4(a), 4(b), and 4(c) show the variation in plasma and urine creatinine levels and creatinine clearance in gentamicin-intoxicated rats treated with the stigma, tepal, and leaf extracts. The findings show that daily gentamicin injection significantly increased plasma creatinine (*p* < 0.001) and significantly decreased urine creatinine clearance (*p* < 0.001) in comparison to normal rats. Daily administration of stigma, tepal, and leaf extracts significantly (*p* < 0.05, *p* < 0.01, and *p* < 0.05, respectively) protected gentamicin-intoxicated rats against the increase in the plasma creatinine level compared to untreated rats (GM group). In addition, the STG and TPL-treated groups showed a significant increase in the urinary creatinine level compared to the GM group. The TPL-treated group showed significant prevention against the decrease in creatinine clearance. But the rats treated with STG and LV revealed no significant effect on clearance compared to the GM group.

### 3.5. The Effect of Gentamicin and *C. sativus* Extracts on Plasma and Urinary Urea

**Figure 5:**
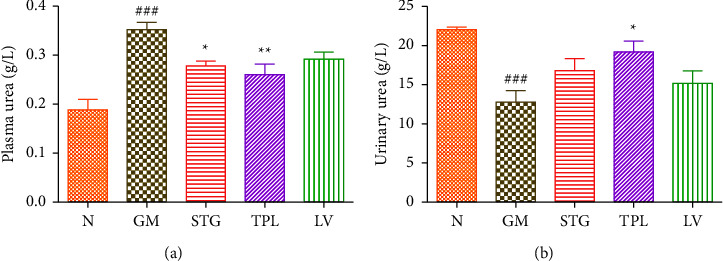
The effect of gentamicin and *C. sativus* extracts from stigmas (50 mg/kg), tepals (250 mg/kg), and leaves (250 mg/kg) on plasma (a) and urine (b) urea in rats. Values were expressed as mean ± SEM. (*n* = 5); ^###^*p* < 0.001 in comparison with the N group;  ^*∗*^*p* < 0.05 and  ^*∗∗*^*p* < 0.01 in comparison with the GM group.

Figures 5(a) and 5(b) represent the variation of plasma and urinary urea levels in gentamicin-intoxicated rats treated with stigma, tepal, and leaf extracts. Rats intoxicated by gentamicin showed a significant (*p* < 0.001) increase in the plasma urea level and a significant (*p* < 0.001) decrease in the urine urea level compared to the normal group. However, STG and TPL treatment significantly reduced the plasma urea level (*p* < 0.05 and *p* < 0.01, respectively) compared with the GM group. LV treatment showed no significant effect on plasma urea levels compared with the GM group. LV administration significantly (*p* < 0.05) increased the urinary urea level compared with the GM group. But administration of STG and LV showed no significant effect compared with the GM group.

### 3.6. The Effect of Gentamicin and *C. sativus* Extracts on Plasma and Urinary Uric Acid

**Figure 6:**
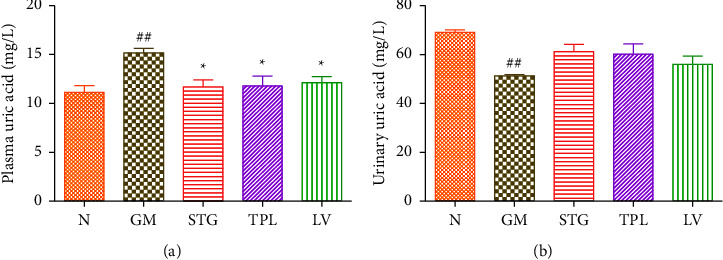
Effect of extracts from stigmas (50 mg/kg), tepals (250 mg/kg), and leaves (250 mg/kg) of *C. sativus* on plasma and urinary uric acid levels ((a) and (b)) in gentamicin-intoxicated rats. Values were expressed as mean ± SEM. (*n* = 5); ##*p* < 0.01 in comparison with the N group;  ^*∗*^*p* < 0.05 in comparison with the GM group.

Figures 6(a) and 6(b) examine the change in plasma and urinary uric acid levels in gentamicin-intoxicated rats treated with STG, TPL, and LV. Gentamicin treatment of rats caused a significant increase in the plasma uric acid level (*p* < 0.01) and a significant decrease in the urinary uric acid level (*p* < 0.01) compared with healthy rats. Administration of STG, TPL, and LV significantly reduced the plasma uric acid level (*p* < 0.05) compared with the GM group. In contrast, treatment with STG, TPL, and LV did not significantly reduce urinary uric acid levels compared with the GM group.

### 3.7. The Effect of Gentamicin and *C. sativus* Extracts on Urinary Protein and Albumin

**Figure 7:**
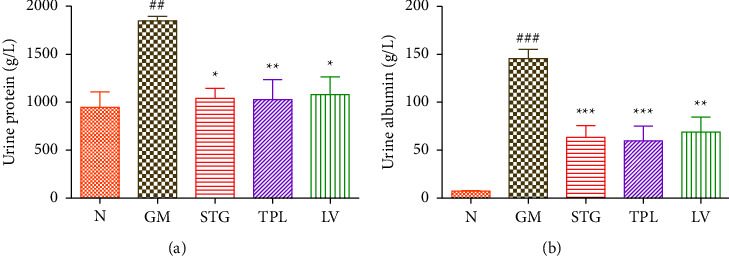
Effect of extracts from stigmas (50 mg/kg), tepals (250 mg/kg) and leaves (250 mg/kg) of *C. sativus* on urinary protein and albumin levels ((a) and (b)) in gentamicin-intoxicated rats. Values were expressed as mean ± SEM. (*n* = 5); ^###^*p* < 0.001 and ^##^*p* < 0.01 in comparison with the control group;  ^*∗*^*p* < 0.05 and  ^*∗∗*^*p* < 0.01 in comparison with gentamicin-intoxicated rats.

The effect of STG, TPL, and LV on urinary protein and albumin levels in rats treated with gentamicin is shown in Figures 7(a) and 7(b). Gentamicin caused a significant increase in urinary protein and albumin levels (*p* < 0.01 and *p* < 0.001, respectively) compared with healthy rats. Administration of STG, TPL, and LV significantly protected against elevation in urinary protein (*p* < 0.05, *p* < 0.01, and *p* < 0.05, respectively) and albumin (*p* < 0.001) levels compared with the GM group.

### 3.8. The Effect of Gentamicin and *C. sativus* Extracts on Plasma Electrolytes (Na^+^, K^+^, and Cl^−^)

**Figure 8:**
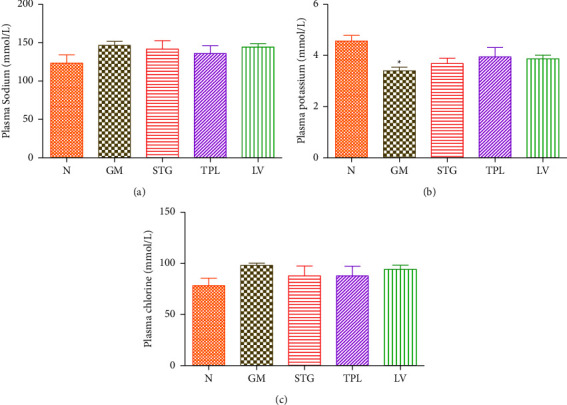
((a), (b), and (c)): The effect of extracts from stigmas (50 mg/kg), tepals (250 mg/kg), and leaves (250 mg/kg) of *C. sativus* on plasma electrolytes (Na^+^, K^+^, and Cl^−^) in gentamicin-intoxicated rats. Values were expressed as mean ± SEM. (*n* = 5); ^#^*p* < 0.05 in comparison with the N group.

The effect of STG, TPL, and LV on plasma sodium, potassium, and chlorine levels in gentamicin-treated rats is shown in Figures 8(a), 8(b), and 8(c). Daily injection for 14 days of gentamicin caused a significant increase in plasma potassium levels (*p* < 0.05) compared with normal rats. Gentamicin treatment did not significantly influence plasma sodium and chlorine levels compared with the GM group. The administration of STG, TPL, and LV did not significantly influence plasma electrolyte levels compared with the GM group.

### 3.9. The Effect of Gentamicin and *C. sativus* Extracts on Lipid Peroxidation (MDA)

**Figure 9:**
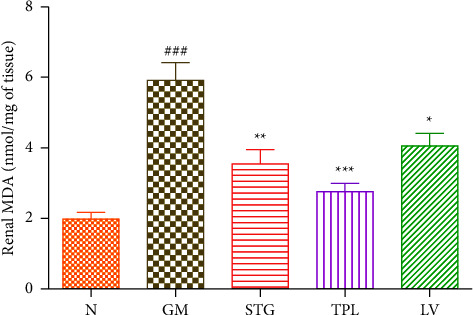
Effect of extracts from stigmas (50 mg/kg), tepals (250 mg/kg), and leaves (250 mg/kg) of *C. sativus* on MDA levels in GM-intoxicated rats. Values were expressed as mean ± SEM. (*n* = 5); ^###^*p* < 0.001 in comparison with the N group;  ^*∗*^*p* < 0.05,  ^*∗*^*p* < 0.01, and  ^*∗∗∗*^*p* < 0.001 in comparison with the GM group.

The impact of the effect of *C. sativus*by-product extracts on lipid peroxidation in treated experimental animals is presented in Figure 9. The intraperitoneal injection of GM caused a significant (*p* < 0.001) elevation of hepatic MDA compared to normal rats. Daily treatment of intoxicated and treated rats with the extracts of stigmas, tepals, and leaves gave a significant reduction (*p* < 0.01, *p* < 0.001, and *p* < 0.05 respectively)

### 3.10. Chemical Composition of *C*. *sativus* Stigmas, Tepals, and Leaves

**Figure 10:**
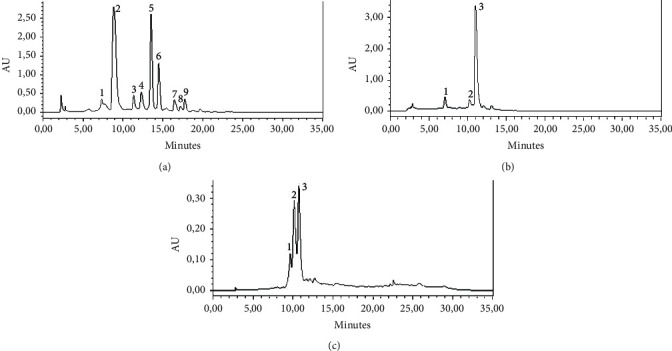
HPLC-DAD chromatograms of *C. sativus* stigmas (a), tepals (b), and leaves (c).

The HPLC-DAD chromatograms and the chemical composition of *C. sativus* stigmas, tepals, and leaves are shown in Figures 10(a), 10(b), and 10(c) and in Table 1 (A, B, and C), respectively. The hydroethanolic extract of stigmas was found to be rich in crocin and picrocrocin (Figure 1(a); Table 1 A). The chemical analysis of the hydroethanolic extract of tepals revealed the presence of isorhamnetin, quercetin, and kaempferol (Figure 1(b); Table 1 (A)). The hydroethanolic extract of leaves is rich in hesperidin, mangiferin, and kaempferol (Figure 1(c); Table 1 (C)).

## 4. Discussion

The results showed that gentamicin-treated rats showed glomerular degeneration, necrosis in Bowmen's capsule, a distinction of Bowman's space, mononuclear cell infiltration, tubular atrophy, tubular dilatation, and cellular desquamation. These histological changes are in agreement with several studies [18, 19]. However, treatment with stigmas, tepals, and leaves preserved kidney morphology at the glomerular and tubular cell level. Indeed, we have shown that intraperitoneal administration of gentamicin significantly reduces body weight, which are consistent with the results found by several authors [20]. According to Hashim and these collaborators, anorexia and increased catabolism are responsible for the decrease in food intake and resulted in weight loss [21]. In addition, damage to tubular cells, involved in renal water reabsorption, leads to dehydration and decreased body weight [22]. An inflammatory response and renal edema may be to blame for the considerable rise in the relative kidney weight seen in the group treated with gentamicin. [23]. This was confirmed by histological sections, which showed apparent renal tubular swelling (Figure 1).

Rats from the gentamicin-intoxicated group (GM group) drank a lot of water and peed a lot. Our results are therefore supported by those of Ogundipe et al. [24]. The polyuria seen during GM treatment is likely caused by the distal convoluted tubules and cortical collecting ducts' inability to reabsorb water from urine, which may be caused by gentamicin's direct effect on the tubules making the tubules less sensitive to ADH (antidiuretic hormone), resulting in difficulty in reabsorbing water [25]. This is confirmed by histology which reveals the presence of damage to the nephrons.

Creatinine has been described as a potent biomarker for clinically evaluating the renal function [26]. The liver and proximal tubules of the kidney release creatinine, which the blood subsequently carries to the muscles. Glomerular filtration and, to a lesser extent, tubular secretion are processes that creatinine goes through. Depending on the source of measurement and the individual muscle mass, creatinine concentrations can change (serum or urine). Our results are confirmed by those of Erdem et al. [27], who reported that gentamicin induced an elevation of plasma creatinine and a decrease in urinary creatinine compared to the control. In addition, they showed that this imbalance is linked to significant kidney histological changes that point to a deterioration in the renal function. An indicator of the glomerular filtration rate is creatinine clearance. Reduced glomerular filtration rate and renal blood flow as a result of increased renal blood vessel constriction or injury to the glomerular capillary endothelium are indicated by decreased creatinine clearance. The considerable reduction in creatinine clearance seen in gentamicin-treated rats suggests both significant tissue damage and reduced glomerular function. Histopathology, which depicts the modification of the glomeruli and the loss of cellular components of the tubules, served as a representation of this. These findings concur with those of Nafiu et al. and Ogundipe et al. [24, 25].

Urea, a product of amino acid metabolism, represents an efficient means of eliminating NH3, which is toxic to the body. The urinary tract is permeable to urea except for the distal convoluted tubule and the initial part of the collecting tube. Reabsorption of urea is passive and takes place mainly in the proximal convoluted tubule: reaching about 50% of the urea filtered at the glomerulus. About 10% of urea molecules are reabsorbed in the collecting tubules near the papilla. In total, 40% of the urea molecules in the glomerular filtration are excreted. Urea is considered an important indicator for examining the renal status. Their elevated concentration in plasma indicates renal failure [28]. According to the findings of our investigation, gentamicin caused a large increase in plasma urea and a considerable fall in urine urea, results that are similar to those of Udapa and Prakash [29].

The breakdown of nucleic acids results in the production of uric acid, on the one hand, and dietary purine compounds on the other. Their excretion is low compared to urea. The distal tubule then secretes it after being completely reabsorbed and filtered in the glomerulus. According to reports, uric acid is a sign that chronic renal disease is developing [30]. We found that gentamicin injection caused a significant elevation in plasma uric acid, a result that is supported by the study of Aiswarya et al. [14].

Proteinuria above the norm indicates renal damage whether tubular or glomerular [31]. The glomerular filtration barrier (GFB) usually does not allow the passage of proteins that are retained by their size and charge. The proximal renal tubule participates in the reabsorption of small proteins that pass this barrier. Our results indicate that gentamicin caused a significant increase in the urinary protein content. These findings are confirmed by those of Govindappa et al. [22].

The amount of albumin filtered under physiological conditions is debated. It is then reabsorbed into the proximal tubule by endocytosis and does not appear in significant amounts in the urine. In the presence of filtration barrier abnormalities or tubular reabsorption abnormalities, increased amounts of albumin and protein may be found in the urine, indicating a pathological condition [32]. In this study, the significant increase in albuminuria following gentamicin injection confirms the nephrotoxic effect of this substance via alteration of the glomerular filtration barriers or via tubular damage. Similar results were obtained by the study of Helmy et al. [33].

Aminoglycosides build up on the proximal tubule membranes, affecting electrolyte reabsorption and secretion, and ultimately leading to a homeostatic imbalance that results in renal failure [25]. The proximal tubules are where the majority of the filtered potassium is reabsorbed. Any morphological change in these tubules lowers potassium reabsorption and, as a result, increases urine output of this electrolyte. According to Govindappa et al. [22], GM causes cell necrosis in the proximal tubules [22]. In this study, a significant reduction in the plasma potassium concentration was observed in GM-treated rats, which may be due to the effect of GM on the proximal tubules. In addition, glomerular dysfunction and impaired tubular reabsorption led to a slight increase in sodium and decrease in potassium and magnesium levels in rat plasma [34]. Plasma potassium ion levels may have decreased as a result of gentamicin due to the drug's stimulation of aldosterone secretion [35].

The end product of polyunsaturated fatty acid peroxidation, malondialdehyde (MDA), is typically utilized as a biomarker of this process [35]. MDA levels in the kidneys of the rats used in this investigation significantly increased after receiving injections of GM; that is supported by several studies [22, 36].

Researchers have reported that aminoglycosides are antibiotics capable of causing nephrotoxicity by producing reactive oxygen species, specifically hydrogen peroxide and superoxide [15, 16]. The reaction between hydrogen peroxide and superoxide can produce the hydroxyl radical, a reactive and unstable radical. As a result of the reaction between H_2_O_2_ and Fe^2+^, this radical is created [37]. Thus, gentamicin-induced nephrotoxicity appears to be largely mediated by this mechanism. Through a variety of mechanisms including DNA damage, protein denaturation, and membrane lipid peroxidation, reactive oxygen species can impair and necrotize cells [38]. Our results indicate that renal MDA levels were significantly attenuated by the administration of tepals, stigmas, and leaf extracts. GM-induced nephrotoxicity in rats may be prevented by using herbal extracts with antioxidant capabilities, according to the studies of Bazm et al. and Khazaei et al. [39, 40]. Also, hydroxyl scavengers such as iron chelators have been shown to prevent GM-induced chronic renal failure [41]. Moreover, we have demonstrated in our previous article that the stigmas, tepals, and leaves of *C sativus* are rich in antioxidant molecules, have a strong antioxidant activity, an important metal chelating ability (fe^2+^ and Cu^2+^), and a significant DNA protective effect [42].

Crocetin esters, picrocrocin, and safranal are moreover the three essential components of saffron, they are primarily in charge of flavor, color, and aroma, respectively [43]. Flavonols in stigma are abundant, and specifically, kaempferol derivatives [44]. *C. sativus* leaves and tepals are abundant in bioactive substances including flavonols. Kaempferol, quercetin, and isorhamnetin have been found to be the primary flavonols in the hydroalcoholic extract of tepals [45]. Will, leaves are rich in hesperidin, mangiferin, and Kaempferol [46]. The nephroprotective effect of major components of stigmas, crocin, and safranal has been confirmed by several studies [47, 48]. Similarly, the nephroprotective effect of kaempferol, quercetin, isorhamnetin, hesperidin, and mangiferin, major components of tepals and leaves, had been noted by various investigations [49–53].

Therefore, the nephroprotective effect found in the present study may be related to these bioactive molecules and their characteristics. Compared to other synthetic antioxidants, which could have negative or undesirable side consequences, these natural antioxidants seem to offer safer substitutes.

## 5. Conclusions

The outcomes of the various experiments supported the necessity of utilizing these by-products. Tested extracts have great therapeutic potential for treating gentamicin-induced renotoxicity. The hydroethanolic tepal and stigma extracts appear to have the most significant nephroprotective effect. In order to create potent dietary supplements or phytomedicines, it would also be highly intriguing to confirm these actions through clinical research.

## Figures and Tables

**Table 1:** Chemical composition of *C. sativus* stigmas (A), tepals (B), and leaves (C).

Peak numbers	Compounds	Retention time (min)	% of areas
(A)			
1	Safranal	7.328	3.44
2	Picrocrocin	8.872	41.39
3	Kaempferol	11.340	4.11
4	Trans-crocin-5	13.503	20.54
5	Trans-crocin-4	14.466	10.03
6	Trans-crocin-3	16.435	2.79
7	Trans-crocin-2	17.176	1.13
8	Cis-crocin-4	17.747	2.60
9	Cis-crocin-3	18.863	0.46

(B)			
1	Isorhamnetin	7.051	6.12
2	Quercetin	10.223	5.72
3	Kaempferol	11.035	76.97

(C)			
1	Hesperidin	9.668	13.54
2	Mangiferin	10.15	41.52
3	Kaempferol	10.721	44.94

Crocin-2: ester-di-(*β*-D-glucosyle)-crocétine; crocin-3: ester-(*β*-D-glucosyle)-(*β*-gentiobiosyle)-crocétine; crocin-4: ester-di-(*β*-D-gentiobiosyle)-crocétine; and crocin-5: ester-(*β*-D-triglucosyle)-(*β*-D-gentiobiosyle)-crocétine.

## Data Availability

All the data used to support the findings of this study are included within the article.
